# A Home-Based Exercise Program Driven by Tablet Application and Mobility Monitoring for Frail Older Adults: Feasibility and Practical Implications

**DOI:** 10.5888/pcd14.160227

**Published:** 2017-02-02

**Authors:** Hilde A.E. Geraedts, Wiebren Zijlstra, Wei Zhang, Sophie L.W. Spoorenberg, Marcos Báez, Iman Khaghani Far, Heribert Baldus, Martin Stevens

**Affiliations:** 1 Center for Human Movement Sciences, University of Groningen, University Medical Center Groningen, Groningen, The Netherlands; 2 Institute of Movement and Sport Gerontology, German Sport University Cologne, Cologne, Germany; 3 Philips Research Europe, Eindhoven, The Netherlands; 4 University of Groningen, University Medical Center Groningen, Department of Orthopedics, Groningen, The Netherlands; 5 University of Trento, Trento, Italy

## Abstract

**Introduction:**

Stimulation of a physically active lifestyle among older adults is essential to health and well-being. The objective of this study was to evaluate the feasibility and user opinion of a home-based exercise program supported by a sensor and tablet application for frail older adults.

**Methods:**

Community-dwelling older adults (aged ≥70 y) living in The Netherlands were recruited in 2014. Participants exercised 3 months with and 3 months without supervision from a remote coach. Feasibility was operationalized as adherence to exercise (percentage of 5 exercise bouts per week completed) and to wearing the sensor (with 70% defined as sufficient adherence) and the number of problems reported. User opinion was measured with a questionnaire addressing ease of use of the technology and opinion on the program.

**Results:**

Twenty-one of 40 enrolled participants completed the trial. Adherence overall was 60.9% (average of 3 bouts per week). Adherence among completers (69.2%) was significantly higher than adherence among dropouts (49.9%). Adherence was sufficient among completers during the 3 months of supervision (75.8%). Adherence to wearing the sensor was 66.7% and was significantly higher among completers than among dropouts (75.7% vs 54.2%). The rate of incidents was significantly lower among completers than among dropouts (0.4 vs 1.2 incidents per participant per week). Connectivity-related incidents were prominent. On a scale of 1 to 5, completers gave ratings of 4.3 (after 3 months) and 4.2 (after 6 months).

**Conclusion:**

A home-based exercise program using novel technology seems feasible when participants are given a stable internet connection. This program shows promise for stimulating physical activity among older frail adults, especially if it offers regular coaching.

## Introduction

The preservation of health and self-reliance in advancing age is increasingly important as the aging population expands (1,2). Older adults generally are insufficiently physically active to maintain health and physical functioning (3,4). Stimulation of a physically active lifestyle among older adults is therefore essential (5).

Older adults often prefer exercising at home (6), but guidance and motivation is necessary and has not been adequate in home-based exercise programs (7). However, the development of internet and novel technology, which can be used to enable remote administration of and guidance in home-based exercise programs, is rising exponentially and could fulfill this need (1,7). Body-worn sensors for measurement of physical activity are being developed on a large scale to accurately and objectively measure daily physical activity and support individually tailored stimulation of physical activity (8–14). Electronic tablets and smartphones that use mobile internet are especially suitable for programs that rely on remote coaches to stimulate adherence to exercise regimens. Text messages, exercise instruction through video contact, and contact with a coach who follows one’s achievements on the internet have been successfully implemented (15–17). Motivational contact with a coach seems to be especially important for adherence (15–17). Internet-based platforms that integrate body-worn sensors and video instructions through a tablet are promising for allowing older adults to exercise independently (18).

Although older adults have traditionally been unwilling and uncomfortable using innovative technology, computer, tablet, and smartphone use among older adults is on the rise. For instance, in 2014, 18% of US adults and 65% of Dutch adults aged 64 or older owned a tablet (19–24). Exercise programs using novel technology for older adults have been reported, although research is necessary on the effective features of these novel technologies and on the potential problems in implementing them in this target age group (18,25,26). The aim of this study was to assess the feasibility and user opinion on independent use of remote novel technology in a home-based exercise program for older adults.

## Methods

This intervention was designed as a prospective cohort study. Participants took part in a 6-month home-based exercise program, using a tablet for exercise instructions and a necklace-worn sensor for daily activity registration. In the first 3 months, which were supervised, participants were contacted weekly by telephone to receive coaching from a human-movement scientist or physiotherapist. These calls addressed motivation, barriers to exercising, and exercise load. During the second 3 months, which were not supervised, participants were not contacted by the coach but could call the coach themselves if they encountered problems. If problems could not be solved by telephone, the coach visited the participant’s home. The exercise component took place from January 2014 through May 2015. The study protocol was approved by the Medical Ethical Committee of University Medical Center Groningen. Details of the study design are provided elsewhere (27).

Participants were community-dwelling older adults living in the province of Groningen, The Netherlands. Most participants lived in remote parts of the countryside, with below-par telephone and internet service. Inclusion criteria were being aged at least 70 years and being able to walk at least 10 meters independently or by using a walking aid. In addition, participants had to be transitionally frail as indicated by a Groningen Frailty Indicator (GFI) score of 4 or 5 (28). The range of the GFI scores is 0 to 15, with higher scores indicating greater frailty. A score of 4 or 5 indicates a minor elevated chance of loss of functionality and heightened disability (28).

Exclusion criteria were physical conditions that hamper safe independent performance of a home-based exercise program or working with a tablet, such as severe visual problems. Participants were recruited from January through November 2014 by means of advertisement, leaflets, and recruitment during meetings of Embrace, a population-based program for integrated elderly care, where community-dwelling older adults could receive information on all aspects of aging (29).

### Technical components

**Necklace-worn sensor.** The sensor was a miniature hybrid sensor containing a 3D microelectromechanical systems (MEMS) accelerometer and a barometric pressure sensor, worn as a necklace. Accelerometer data were sampled at 50 Hz with a range of 8g; barometric data were sampled at 25 Hz. Accelerometer data were used to identify activities, such as sitting, standing, and walking (10,21,22). A micro-SD card was used for storage and exchange of data. The sensor weighs 30 g and measures 55 mm by 25 mm by 10 mm (Philips Research Eindhoven). Data were transferred and batteries were recharged automatically each night when the sensor was connected to the tablet by USB cable. Users wore the sensor during the day, and adherence to wearing the sensor was tracked by the tablet by means of the plug-in and plug-out time of the sensor.

**Tablet.** A tablet (Latitude 10, Dell; Windows 8 operating system, Microsoft Corp) was given to each user; it gave exercise instructions by means of videos and remote feedback. Functionality of the tablet was adjusted to independent older adult use, by designing menus to be as simple as possible by reducing menu options, providing an automatic restart when the tablet was shut down, and enlarging icons. Internet connection was provided through a mobile internet card with a 3G, 4G, or Wi-Fi connection. The exercise program was provided by an internet-based application running on a remote web server.

**Exercise program.** The strength and balance exercise program was provided in 18 levels, starting with 10 minutes and increasing to about 45 minutes. Participants were asked to exercise 5 times per week. Participants were able to choose their own level. Exercises were shown in videos. Each level had a different video showing a full exercise bout. After completing a video, a tailored motivational message as well as sensor-registered graphical feedback on daily activity during previous days were provided. The program is described in detail elsewhere (27).

### Evaluation methods

**Adherence. **Adherence to the program was calculated according to completion of the planned exercise bouts as indicated by watching the exercise videos. For example, if a participant completed 3 of 5 bouts in a week, the adherence rate was calculated as 60%. Adherence to wearing the sensor was calculated according to the number of days the sensor was worn with successful collection of data as registered by the sensor. Scheduled holidays were excluded from analysis. Adherence was considered sufficient when adherence to the program and wearing of the sensor exceeded 70%.

**Technical and operational feasibility.** An inventory was made of problems encountered by participants during the program. All telephone calls and home visits other than scheduled contacts were cataloged, and the reasons for the calls or visits were noted. Problems encountered by participants were divided into 3 categories: technology-related, connectivity-related, and participant-induced. Technology-related incidents were, for example, malfunctioning of cables or a defective sound card. Connectivity-related incidents were related to poor internet coverage or server downtime. Participant-induced incidents were, for example, opening the incorrect web pages. The number of incidents was assessed as well as their density (mean number of incidents per participant per week).

**Determinants of participation. **Factors that might have influenced participants’ ability to independently perform a home-based exercise program using novel technology were assessed by means of a questionnaire. Questions asked about age, sex, health conditions, and previous and current use of computers and smartphones. Height and weight were measured, and body mass index was calculated. Participants also completed the 15-item GFI (28). Previous and current use of a personal computer was assessed by means of a multiple-choice question with the answer categories of “never used before,” “occasionally,” or “daily, now or in the past.” Smartphone ownership was assessed by tabulating dichotomous responses of “own a smartphone” or “do not own a smartphone.”

**User evaluation. **User evaluation was performed by means of a questionnaire (an adapted version of the SensAction-AAL (Sensing and Action to Support Mobility in Ambient Assisted Living) participant evaluation form [30]) completed by participants after the supervised period and again after the unsupervised period. The questionnaire addressed ease of use of the tablet and sensor, frequency of contact and help from the coach, and trust in the correct functioning of the devices. Answer categories ranged on a Likert scale from 0 (“do not agree at all”) to 5 (“fully agree”). A higher score indicated a more positive opinion. Dropouts were contacted by telephone and asked a single question after they ended their participation: “Rate the program and technology with a mark between 1 and 5, 1 being very ill-performing and not enjoyable and 5 indicating very well-performing and enjoyable.”

### Statistical analysis

We tabulated data on the characteristics of the study sample, including information on sex, age, GFI score, body mass index, computer experience, smartphone ownership, and type of internet service used. We tabulated data for all participants combined and then compared participants who completed the program (completers) with participants who dropped out (dropouts). We used 1-way analysis of variance (ANOVA) and independent-samples *t* tests to examine differences between completers and dropouts. We then measured the mean (standard deviation [SD]) number of days in the program, program adherence overall (as an average percentage), average program adherence during the first 3 months, average program adherence during the second 3 months, and adherence to wearing the sensor. We compared these data between completers and dropouts (using independent-samples *t* tests to compare differences) and by type of internet service used (using 1-way ANOVA) to compare differences). We examined the number and density of incidents overall and by category, and we compared data between completers and dropouts and by type of internet service. Finally, we examined data from the evaluation questionnaires, comparing the responses of completers and dropouts. We used SPSS 22.0 (IBM Corp) for all analyses. Significance was set at an α level of ≤.05.

## Results

Forty transitionally frail (mean [SD] GFI score, 4.4 [0.5]) and independently living adults participated; 15 were men. Mean age at intake was 81 (SD, 4.6) years. All participants had 1 or more chronic conditions or debilitations, most commonly heart failure, diabetes, and leg injuries. Twenty-five participants had previous experience with a tablet or laptop, of whom 21 used such a device daily. One participant owned a smartphone (Table 1).

**Table 1:** Characteristics of Participants in a Study to Evaluate the Feasibility and User Opinion of a 6-Month Home-Based Exercise Program, Groningen, The Netherlands, 2014

Variable	All (N = 40)	Completers (n = 21)	Dropouts (n = 19)	*P* Value^a^
**Sex, n**				
Male	15	8	7	.90
Female	25	13	12	
**Age, mean (SD), y**	81 (4.6)	80 (4.7)	83 (4.2)	.06
**Groningen Frailty Indicator, mean (SD) score^b^**	4.4 (0.5)	4.4 (0.5)	4.5 (0.5)	.66
**BMI^c^, mean (SD)**	27.9 (4.1)	28.1 (4.4)	27.6 (3.7)	.75
**Computer experience^d^, n**				
Yes	25	18	7	<.001
No	15	3	12	
**Smartphone owner, n**				
Yes	1	1	0	.04
No	39	20	19	
**Type of internet service, n**				
3G	17	4	13	.04
4G	11	6	5	
Wi-Fi	12	11	1	

Abbreviations: BMI, body mass index; SD, standard deviation.

a *P* values for difference between completers and dropouts determined by independent-samples *t* test.

b Range of score for Groningen Frailty Indicator is 0 to 15; a score of 4 or 5 indicates a minor elevated chance of loss of functionality and heightened disability (28).

c Calculated as weight in kilograms divided by height in meters squared.

d Determined by asking the question “Do you have prior experience with computer, tablet, or smartphone?”

### Adherence

Twenty-one participants completed the program. Of the 19 who dropped out, 11 did because of internet reception problems, 5 did because of medical reasons not related to the program, 2 did because of illness of their spouse, and 1 participant died. Sixteen dropouts quit during the first 3 months. Among all participants, average adherence to exercising 5 times per week was 60.9% (3 bouts per week). Adherence was 69.2% (≈3.5 bouts per week) among completers and 49.9% (2.5 bouts per week) among dropouts (*F* = 0.08; *P* = .05). Adherence during the first 3 months (supervised) differed significantly between completers (75.8%) and dropouts (49.3%) (*F* = 0.05; *P* = .01). During the second 3 months (unsupervised), adherence was 62.4% among completers and 40.5% among dropouts (*F* = 2.30; *P* = .44). Overall adherence among participants was 56.8% for those using 3G, 60.3% for those using 4G, and 64.9% for those using Wi-Fi (*F* = 0.14; *P* = .90). Completers had an adherence of 75.7% for the daily wearing of the sensor, whereas dropouts had a significantly lower adherence of 54.2% (*F* = 1.62; *P* = .04). We found no other significant differences in adherence rates (Table 2).

**Table 2:** Adherence Rates^a^ in a Study to Evaluate the Feasibility and User Opinion of a 6-Month Home-Based Exercise Program, Groningen, The Netherlands, 2014^b^

Variable	All (N = 40)	By Program Completion			By Type of Internet Service			
		Completers (n = 21)	Dropouts (n = 19)	*P* c	3G (n = 17)	4G (n = 11)	Wi-Fi (n = 12)	*P* d
No. of days in intervention (SD)	126.2 (86.8)	202.6 (10.8)	45.7 (49.6)	<.001	70.9 (76.0)	120.6 (90.5)	188.4 (57.7)	.04
Adherence to exercise program	60.9 (32.5)	69.2 (32.2)	49.9 (30.4)	.05	56.8 (31.9)	60.3 (37.5)	64.9 (32.1)	.90
Adherence to exercise program during 3 months of supervised exercise^e^	64.3 (32.1)	75.8 (29.2)	49.3 (30.3)	.01	56.3 (30.7)	56.6 (30.2)	78.8 (33.4)	.35
Adherence to exercise program during 3 months of unsupervised exercise^f^	60.5 (40.6)	62.4 (41.9)	40.5 (18.2)	.44	52.3 (45.3)	75.6 (50.4)	50.6 (34.5)	.47
Adherence to wearing the sensor^g^	66.7 (30.7)	75.7 (27.7)	54.2 (31.2)	.04	62.6 (31.8)	83.7 (23.0)	60.8 (31.8)	.21

a Adherence rates were calculated according to completion of the 5 exercise bouts scheduled each week. For example, if a participant completed 3 of 5 bouts in a week, the adherence rate was calculated as 60%.

b All values are mean (standard deviation) percentage unless otherwise indicated.

c *P* values for differences between completers and dropouts determined by independent-samples *t* test.

d *P* values for differences between type of internet service determined by 1-way analysis of variance.

e First 3 months of the 6-month program were supervised by a remote coach.

f Second 3 months of the 6-month program were not supervised by a remote coach.

g Adherence to wearing the sensor was calculated according to the number of days the sensor was worn with successful collection of data as registered by the sensor.

### Technical and operational feasibility

The total number of incidents was 249; the mean density was 0.8 incidents per participant per week. Density was significantly different between completers (0.4) and dropouts (1.2) (*F* = 7.55; *P* = .01). Of the 249 incidents, 109 were technology-related; these mainly concerned disconnection of the sensor and the tablet, causing the activity data not to be shown. One hundred and eleven incidents were connectivity-related; all were related to internet instability. Twenty-nine incidents were participant-induced; these calls mainly concerned accidental removal of the button for the application from the app menu and opening too many screens. The density for each incident type differed significantly between completers and dropouts (Table 3).

**Table 3:** Number and Density^a^ of Incidents^b^ in a Study to Evaluate the Feasibility and User Opinion of a 6-Month Home-Based Exercise Program, Groningen, The Netherlands, 2014

Variable	All (N = 40)	By Program Completion			By Type of Internet Service			
		Completers (N = 21)	Dropouts (N = 19)	*P* c	3G (n = 17)	4G (n = 11)	Wi-Fi (n = 12)	*P* d
**Technology-related**								
No.	109	80	60	.04	22	29	52	.01
Density	0.2 (0.5)	0.2 (0.2)	0.2 (0.4)		0.2 (0.3)	0.2 (0.4)	0.2 (0.4)	
**Connectivity-related**								
No.	111	51	29	.001	50	25	27	.001
Density	0.4 (0.3)	0.2 (0.3)	0.6 (0.5)		0.7 (0.5)	0.2 (0.4)	0.2 (0.4)	
**Participant-induced**								
No.	29	22	7	.02	6	7	9	.88
Density	0.1 (0.1)	0.05 (0.08)	0.1 (0.2)		0.03 (0.1)	0.1 (0.2)	0.05 (0.1)	
**Total**								
No.	249	153	96	.01	78	61	86	.01
Density	0.8 (0.8)	0.4 (0.5)	1.2 (0.8)		1.3 (0.8)	0.4 (0.3)	0.4 (0.7)	

a Mean (standard deviation) number of incidents per participant per week.

b Problems encountered by participants were divided into 3 categories: technology-related (eg, cable malfunction), connectivity-related (eg, poor internet coverage), and participant-induced (eg, opening incorrect web pages).

c *P* values for differences in density between completers and dropouts determined by independent-samples *t* test.

d *P* values for differences between type of internet service determined by 1-way analysis of variance.

Incident density among 3G users (1.3 incidents per participant per week) was significantly higher than density among 4G users (0.4) or Wi-Fi users (0.4) (*F* = 6.49; *P* = .01). In particular, connectivity-related incident rates were higher among 3G users (0.7) than among 4G users (0.2) or Wi-Fi users (0.2) (*F* = 4.44; *P* = .001) (Table 3).

In the first 3 months of the program, during which participants were contacted weekly by telephone, 216 telephone calls were made. Each called lasted about 1 or 2 minutes when no additional motivation or technological assistance was needed, or 216 to 432 minutes for the 3 supervised months. During 40 of these calls, coaches used motivational strategies. Motivational discussions lasted between 2 and 5 minutes each, or 80 to 200 minutes for the 3 supervised months. In 23 of these calls, participants were advised to adjust their training load by training on a higher or lower level. In the other 17 calls, participants received feedback on their adherence when adherence seemed to be below par. No participants called coaches during the unsupervised part of the intervention.

### User evaluation

The average score on the user evaluation questionnaire among completers was 4.3 (SD, 0.4; range, 0–5) after 3 months and 4.2 (SD, 0.2; range 0–5) after 6 months. Eighteen of 21 participants indicated a preference for receiving weekly telephone calls (ie, supervision) rather than exercising independently (no supervision).

Eleven of the 19 dropouts responded to the user evaluation questionnaire. The mean score on the user evaluation questionnaire was 2.0 (SD, 0.9; range, 0–4). Of these 11 dropouts, 4 had not left the program because of internet reception problems and rated the program significantly more positively than the 7 dropouts who left because of internet reception problems (2.8 [SD, 1.0] vs 1.6 [SD, 0.5]; *P* = .03).

## Discussion

Our study provides insight into the feasibility of a home-based exercise program using a body-worn sensor and a tablet application. Mean adherence to the exercises was 60.9%, which did not reach our goal of 70%. However, adherence among completers reached 75.8% during the supervised part of the intervention. That adherence decreased to 62.4% among completers during the unsupervised part indicates that weekly coaching is an important influence on exercise levels. This finding is in line with earlier findings showing the importance of coaching (15–18). Adherence to wearing the sensor fell short of the 70% goal among all participants (66.7%) but surpassed the goal among completers (75.7%). Our adherence rates, however, are lower than those reported in previous studies (18). The difference in rates between our study and previous studies can be explained by the longer intervention time in our study. Long trials lead to higher rates of nonadherence than do short trials (18). However, in our study, adherence was probably mostly influenced by stability of the internet system, which was often compromised by connectivity issues.

The use of mobile internet connections has drawbacks, illustrated by the high rate of connectivity-related incidents. In semi-rural areas such as Groningen, levels of 3G coverage are often low. Internet pages not loading caused discontentment among participants; 7 of the 11 dropouts who responded to the user questionnaire dropped out because of 3G malfunction. Because older adults living in remote areas that lack convenient exercise facilities might especially benefit from remotely coached home-based exercise programs (18), it is important to solve the problem of internet malfunction. One solution was to switch 3G-network users to a different internet provider with a higher rate of coverage and a 4G network. After switching to a new provider and from 3G to 4G, the problem with internet reception was solved; adherence increased, and the dropout and incident rates among 4G users decreased. Another solution to the problem of mobile internet malfunction is using a Wi-Fi connection. None of the 12 participants using Wi-Fi dropped out because of connectivity problems, and connectivity-related incidents were less prevalent among Wi-Fi users than among 3G or 4G users. A stable, reliable internet connection is a major influence on adherence to an internet-supported, home-based exercise program.

Participants who completed the intervention were enthusiastic about the technology used in the intervention, giving a mean rating of 4.3 of 5 in the appreciation of the tablet, sensor, coaching, and exercise program. However, the questionnaire was administered after 3 months and after 6 months, and therefore dropouts did not complete it. This selection bias skewed results toward the positive. The 11 dropouts who answered the user questionnaire gave low ratings (mean 2.0 of 5), indicating that they were not pleased by the performance of the application. Of these 11 participants, 7 dropped out primarily because of internet instability. The 4 participants who did not drop out because of internet instability had a significantly more positive view of the application than those who did. Internet instability was a key determinant of user opinion among dropouts.

The demand on coaches in this intervention was small when technology was stable: only 1 to 2 minutes per participant per week or 2 to 5 minutes when participants needed motivational coaching. The additional effort of weekly telephone calls is, therefore, a small but necessary investment. It can be expected that when ideal circumstances prevail (a stable internet connection and regular coaching), adherence to the exercises and wearing of the sensor would reach 70%.

The design of our study has several strengths. First, the program lasted 6 months, a fairly long period for an exercise intervention and long enough to indicate long-term adherence. Second, 40 participants in an extensive intervention like ours is a substantial number of participants. Third, with a mean age of 80.8 years and diverse medical conditions, participants were representative of a general sample of older adults. Participants were also diverse in their experience with technology, providing information on feasibility for both beginners and more experienced users.

Our study has at least one limitation: the problems with internet instability. The unreliable internet connection caused a high drop-out rate and probably caused the feasibility of the application to be underrated.

A home-based exercise program using novel technology for older adults seems feasible. Adherence was sufficient among the completers in the coached part of the intervention, indicating that regular coaching is a positive influence on adherence. Dropout, adherence, and user opinion were strongly influenced by stability of the internet connection and system. The demand on the coaches’ time was low when participants had a stable internet connection. The adults completing the program had a positive opinion of using the technology in performing the exercise program.
